# New Butyroside D from Argan Press Cake Possess Anti-Melanogenesis Effect via MITF Downregulation in B16F10 and HEM Cells

**DOI:** 10.3390/ijms232416021

**Published:** 2022-12-16

**Authors:** Meryem Bouhoute, Yhiya Amen, Meriem Bejaoui, Aprill Kee Oliva Mizushima, Kuniyoshi Shimizu, Hiroko Isoda

**Affiliations:** 1 Alliance for Research on the Mediterranean and North Africa (ARENA), University of Tsukuba, Tsukuba 305-0006, Japan; 2 Department of Agro-Environmental Sciences, Faculty of Agriculture, Kyushu University, Fukuoka 819-0395, Japan; 3 Department of Pharmacognosy, Faculty of Pharmacy, Mansoura University, Mansoura 35516, Egypt; 4 Research and Development Center for Tailor-Made QOL Program, University of Tsukuba, Tsukuba 305-8550, Japan; 5 Faculty of Life and Environmental Sciences, University of Tsukuba, Tsukuba 305-8577, Japan

**Keywords:** hyperpigmentation, Butyroside D, MAPK, cAMP, Wnt, MITF

## Abstract

Hyperpigmentation is a skin condition where patches of skin become darker in color due to excess melanin production upon UV exposure leading to melasma, which are lentigines or post inflammatory hyperpigmentation that psychologically affecting a great number of people. The present study investigates the anti-melanogenic effect of Butyroside D and the underling mechanism. After the confirmation of the non-cytotoxic effect of Butyroside D on B16F10 cells, we proceeded with analyzing the impact of the treatment at low and high concentration (i.e., 0.2 μM and 2 μM) using gene profiling analysis and examined the differentiation in gene expression. Our results identify cyclic adenosine monophosphate (cAMP), Wnt/β-catenin and Mitogen-Activated Protein Kinase (MAPK) signaling pathways to be downregulated upon treatment with Butyroside D. These pathways were targeted to further validate the effect of Butyroside D on membrane receptors melanocortin 1 receptor (MC1R) and receptor tyrosine kinase (c-Kit), related microphthalmia-associated transcription factor (MITF) and consequently tyrosinase (TYR), and tyrosine-related protein-1 (TYRP-1) that were all shown to be downregulated and, therefore, leading to the repression of melanin biosynthesis. Finally, the anti-melanogenic effect of Butyroside D was confirmed on human epidermal melanocytes (HEM) cells by inhibiting the activation of cAMP pathway generally mediated through α-melanocyte-stimulating hormone (α-MSH) and MC1R. Overall, this study suggests the potential applicability of this purified compound for the prevention of hyperpigmentation conditions.

## 1. Introduction

The process of melanin synthesis and distribution, also called melanogenesis, is initiated within the melanocytes, which are pigment producing dendritic cells [1]. Originally, melanocytes precursors, known as the melanoblasts, are unpigmented cells that originate from embryonic neural crest cells before migrating to the basal layer of skin epidermis and hair follicles [2,3,4]. The melanin is synthesized and stored in the melanocytes within lysosome-like organelles called the melanosomes before their transfer to the neighboring keratinocytes [5]. Specialized enzymes and structural proteins are, however, needed to produce melanin, including tyrosinase (TYR), tyrosinase-related protein 1 (TYRP1), and dopachrome tautomerase (DCT) or TYRP2 [6]. These critical enzymes that affect the quantity and quality of melanin are under the influence of microphthalmia-associated transcription factors (MITF) that are involved in the survival as well as the proliferation of melanocytes [7]. Generally, the oxidation of L-tyrosine and/or L-dihydroxyphenylalanine (L-DOPA) to dopaquinone (DQ) initiates melanin synthesis and the resulting quinone serve as a substrate for eumelanin (brown/black) and pheomelanin (red/yellow); the eumelanin/pheomelanin ratio and the size of melanosomes leads to skin color variation [8,9,10]. TYR performs a rate limiting activity because all further reactions can proceed at physiological pH condition; however, TYRP-1 and TYRP-2 catalyze the conversion of DQ into melanin [8,11]. Several intracellular signaling pathways modulate melanogenesis; these include cAMP/PKA via MC1R/α-MSH, PI3K/Akt signaling, Wnt/β-catenin signaling, SCF/c-kit mediated signaling, nitric oxide (NO) or cytokines, MAPK cascade, and autophagy-mediated associated mechanisms [12]. In addition, DNA-damaging environmental influences, mainly ultraviolet radiation (UVR), toxic chemicals or other factors such as antioxidant enzyme, initiate the regulatory system leading to melanin synthesis and maintain epidermal homeostasis to protect the skin from photo-aging and DNA damage [13]. Globally, melanocyte’s function and pigment synthesis are genetically regulated by around 125 distinct genes; moreover, these processes are further influenced by differences in the post-transcriptional regulation of melanogenesis-related genes [14].

Nonetheless, excessive production and accumulation of melanin induce epidermal hyperpigmentation characterized by an increased activation of melanocytes that could lead to skin diseases such as melasma, freckles, and melanosis and solar lentigines with an enormous impact on patients’ psychology [15]. Several researchers have reported the utilization of natural extracts to inhibit melanin production [16,17,18,19]. Moreover, autophagy also plays an important role in melanin homeostasis and melanogenesis, and several natural compounds showed depigmentation potential through autophagy [20]. However, the complex mixture in crude extracts fail to identify a single compound responsible for the investigated effect and rather hypothesize the action of several molecules in the extract [21,22]. Therefore, it is important to develop and characterize a safe and effective skin whitening agent that is able to significantly reduce hyperpigmentation disorder in its pure form as an attempt to identify the causal effect of the crude extract.

*Agrania spinosa,* an endemic species of Morocco, was found to be a source of several extracts with confirmed biological activities [23]. Those extracts are derived from different parts of the plant, such as leaves, pulp, shell or even nut residue recovered from oil extraction identified as Argan press cake [24,25,26,27,28,29,30]. The increase of interest in the cosmeceutical application of Argan derivatives deployed efforts to bring its noble composition to proper use [31]. Among those, the characterization of its extracts sheds light on the rich composition of its secondary metabolites [32,33,34]. Specifically, polar saponins were isolated from Argan press cake using 50% aqueous-ethanolic extract and identified Arganine A, B, C, D, E, F, Butyroside C, and Tieghemelin and are derived from protobassic acid or 16-α hydroxy-protobassic acid. Among these saponins, Butyroside D, 3-O-glucuronopyranosyl 16-hydroxyprotobassic acid 28-O-apiofuranosyl-1-3-xylopyranosyl-1-4-rhamnopyranosyl-1-2-arabinopyranoside was identified in *Argania spinosa* press cake [35], following its identification in *Madhuca butyracea* seed [36]. However, little to nothing is known about the actual biological activity of this saponins without being part of the crude complex extract mixture, and an investigation of Butyroside D pure saponin is still lacking. In fact, the crude extract from Argan press cake was found to significantly decrease B16F10 melanin synthesis through the downregulation of MITF leading to a decrease in the level of expression of melanogenic enzymes Tyr, Tyrp1, and Tyrp2 [29].

In this study, the molecular mechanism underlying the anti-melanogenesis potential effect of Butyroside D purified from Argan press cake ethanolic extract was investigated. Microarray analysis was used to identify key regulated genes and pathways upon the treatment of B16F10 cells with this compound. Thus, the study identifies cAMP, MAPK, and Wnt/β-catenin signaling pathways to be downregulated when the cells are subjected to Butyroside D. Consequently, melanogenesis key related gene expression levels were validated to further confirm the effect of Butyroside D on pigmentation upstream effectors on B16F10 murine cells and on human epidermal melanocytes. To the best of our knowledge, this study is the first describing the anti-melanogenesis effect of purified Butyroside D.

## 2. Results

### 2.1. Butyroside D Structure Identification

**Figure 1:**
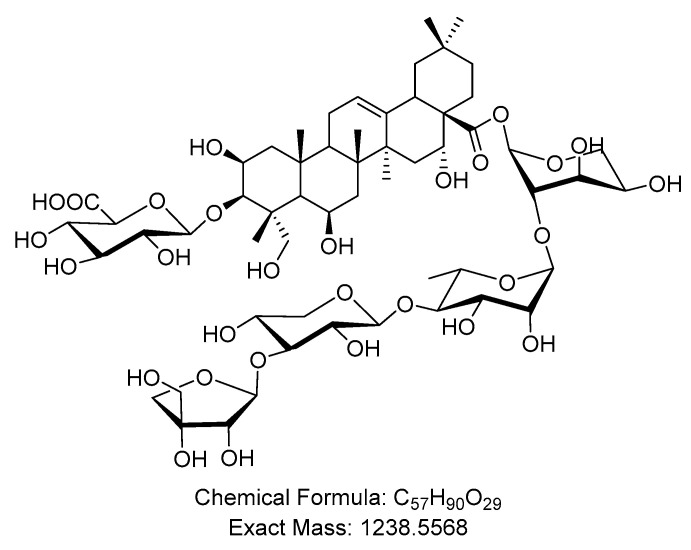
Chemical structure of Butryoside D, chemical formula of C_57_H_90_O_29_ and an exact mass of 1238.5568.

Butyroside D was isolated as a white amorphous powder,$\;\left[\alpha \right]\begin{array}{c} 25\\ D \end{array}$ − 22.4 (*c* 0.86, EtOH). HR-ESI-MS displayed a deprotonated molecular ion peak at *m*/*z* 1237.5513 [M − H]^−^, calculated (1237.5490) (Supplementary Data). The investigation of its NMR data (Supplementary Data) revealed that it is a 3,28-di-*O*-bisdesmoside of 16α-hydroxy protobassic acid. The ^1^H-NMR spectrum showed six singlets integrated for three protons each, attributable to six methyl groups at δ_H_ 0.86, 0.95, 1.02, 1.28, 1.31, and δ_H_ 1.60, assigned for H-29, H-30, H-26, H-24, H-27, and H-25, for the aglycone moiety. One set of a three-proton doublet was observed at δ_H_ 1.27 (*d*, *J* = 5.8 Hz) indicated the presence of a deoxyhexopyranosyl unit. The ^1^H-NMR spectrum further displayed one oxygenated proton at δ_H_ 3.57 (br. *m*), assigned for H-3, as evidence for the glycosylation at C-3. An olefinic proton at δ_H_ 5.39 (t, *J* = 3.8 Hz) was assigned for H-12. Five anomeric protons were clearly observed in the ^1^H-NMR spectrum at δ_H_ 4.49 (d, *J* = 7.8 Hz, GlcA H-1), δ_H_ 4.54 (d, *J* = 7.4 Hz, Xyl H-1), δ_H_ 5.09 (br. *S*, Rha H-1), δ_H_ 5.22 (d, *J* = 2.8 Hz, Api H-1), and δ_H_ 5.57 (d, *J* = 4.0 Hz, Ara H-1). This data suggests the presence of a β-glucuronic acid unit, a β-Xyl unit, α-Rha unit, a β-Api unit, and an α-Ara unit. The analysis of obtained NMR spectral data were consistent with those published for Butyroside D [36]. Thus, the compound was identified as Butyroside D as represented in Figure 1.

### 2.2. Butyroside D Did Not Show a Cytotoxic Effect in B16F10

**Figure 2:**
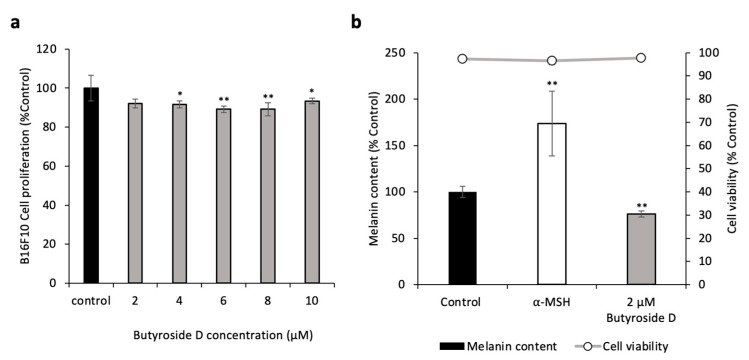
Butryoside D has no cytotoxic effect in B16F10 and inhibit melanin production in B16F10 cells. (**a**) MTT assay was performed to assess the cell proliferation of Butryoside D-treated B16F10 cells varying concentration (2, 4, 6, 8, and 10 μM). (**b**) The intracellular melanin content of B16F10 cells were assessed. B16F10 cells were treated with 2 μM Butryoside D or α-MSH as positive control, for 48 h. Results are presented as mean ± SD (*n* = 3). Statistical analysis was performed using one-way ANOVA where asterisks denote significant differences between the control and test groups being * *p* < 0.05 and ** *p* < 0.01.

First, we evaluated the effect of Butryoside D in B16F10. Therefore, cells were cultured and treated with various concentrations of the sample, then MTT assay was performed. Figure 2a confirms that at low concentrations, Butyroside D did not exert any toxicity to B16F10 cells. However, upon increasing the concentration of the treatment, the pure compound slightly altered the cells viability. Thus, further experimental design focused on the lowest concentration of 2 μM.

### 2.3. Butyroside D Reduced the Melanin Content in B16F10

The melanin assay was conducted to determine the effect of Butyroside D on melanin production using B16F10 widely used in pigment cell studies as these cells can highly produce melanin without further stimulation from α-MSH [37]. Hence, cells were treated with only medium (negative control), 2 µM Butyroside D, or 0.1 µM α-MSH (positive control) for 48 h before conducting the assay. Figure 2b shows a significant reduction of melanin content in B16F10 cell upon treatment with 2 µM Butyroside D, as the melanin concentration value was recorded to 76% compared to the control. The α-MSH positive control group showed an increase in melanin production up to 200%. Interestingly, this effect was observed without decreasing the cell viability for all treatments (Figure 2b). This result suggests that Butyroside D inhibited the melanogenesis in B16F10 cells.

### 2.4. Global Gene Profiling Analysis of Butyroside D in B16F10 Cells

**Figure 3:**
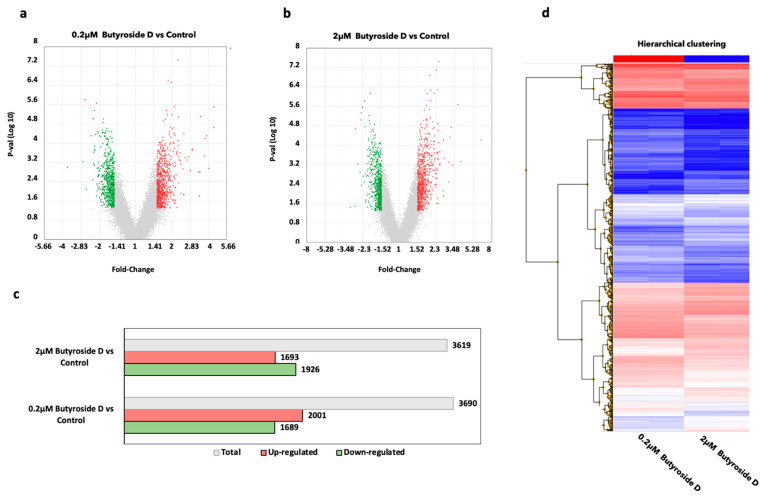
Butryoside D promoted differential expression of genes in B16F10 cells. (**a**,**b**) Volcano plots displaying the DEGs regulated between 0.2 and 2 μM Butryoside D versus control, respectively. The *x*-axis represents the fold change of the DEGs whereas the *y*-axis represents the log_10_ (*p*-value). The green dots represent the downregulated genes, red dots are for the upregulated genes, and the gray dots are the unreguated genes. (**c**) Bar graph showing the total number of regulated DEGs by 0.2 and 2 μM Butryoside D. DEGs were selected according to the fold-change criteria of >1.5 and <−1.5. The *x*-axis represents the number of DEGs and the *y*-axis shows the groups. Red bar indicates the upregulated genes, green bar indicates the downregulated genes, and gray bar represents the total number of DEGs regulated in each group. (**d**) The heatmap of DEGs following the hierarchical clustering analysis. The x-axis shows the sample groups which are 0.2 and 2 μM Butryoside D while the *y*-axis represents the DEGs. Red bars represent the upregulated genes and blue indicates downregulated genes.

A microarray analysis was performed to further investigate the molecular mechanism behind the effect of Butyroside D on B16F10 cells. Thus, the experiment was assessed using RNAs extracted from negative control, 0.2 μM, and 2 μM Butyroside D. An intensity value of total gene probe set after treatment with the samples compared to the untreated cells is displayed in volcano plots in Figure 3a,b. Probes with a threshold of fold change ≥ ±1.5 and *p* ≤ 0.05 were considered as differentially expressed genes (DEGs). Moreover, the volcano plots of the treatment against the control reveals the extent of FC and relative p-values after data normalization using the Expression Console Software (Figure 3a,b). The data revealed that in the case of 0.2 µM Butyroside D vs. the control, 3690 genes were differentially expressed, of which 2001 DEGs were up-regulated and 1689 DEGs down-regulated, whereas in the 2 µM Butyroside D vs. the control, 3690 genes were differentially expressed with 1693 up-regulated and 1926 down-regulated (Figure 3c). In addition, the two treatment conditions were subjected to a comparison using hierarchical clustering, and the result showed similar tendencies in the affected genes between the two concentrations of the sample (Figure 3d).

### 2.5. Butyroside D Downregulated Melanogenesis-Associated Genes in B16F10 Cells

**Figure 4:**
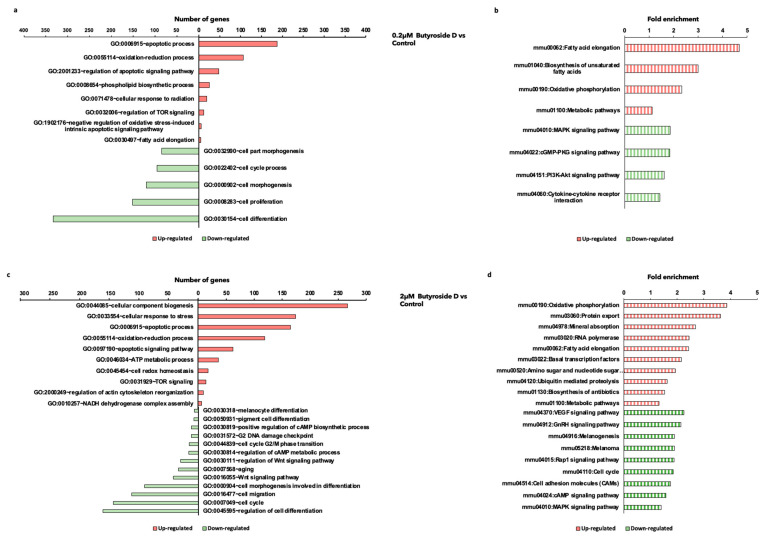
Butryoside D modulated biological processes and pathways related to melanogenesis. Summary of the top (**a**) up- and downregulated gene ontologies (GO) and (**b**) KEGG pathways stimulated by 0.2 μM Butryoside D. Summary of the top (**c**) up- and downregulated gene ontologies (GO) and (**d**) KEGG pathways stimulated by 2 μM Butryoside D. The red bars represent the upregulated genes and green bars represents the downregulated genes. The GO graph *x*-axis indicates the number of genes, and the *y*-axis represents the GOs. KEGG pathway graph *x*-axis reflects the fold change of the pathways indicated in the *y*-axis.

Next, the most regulated biological process (BPs) pathways were determined applying gene ontology (GO) and KEGG pathways analysis of DAVID and GSEA under the conditions qualifying a *p*-value ≤ 0.05 and containing more than two genes in each set. From the viewpoint of downregulated genes, GO analysis revealed an enrichment in cell differentiation, proliferation, morphogenesis, and cell cycle process from a low concentration treatment 0.2 μM Butyroside D (Figure 4a). Furthermore, upon increasing the concentration of the treatment to 2 μM, additional GOs related to cell migration, melanocytes and pigment cell differentiation, cell cycle, regulation of cAMP metabolic process, Wnt signaling pathway, and aging were significantly inhibited (Figure 4c). It is well known that the melanocytes are the pigment producing cells, in which the differentiation further stimulates their activity [38], whereas aging causes cells an unbalance and function distribution [39]. Thus, 2 µM Butyroside D reduced melanogenesis activity without altering melanocyte life cycle.

The KEGG pathways analysis revealed that the low treatment concentration downregulated MAPK, cGMP-PKG, and PI3K-Akt signaling pathways. In addition, the higher concentration further downregulated the above mentioned pathways, along with reducing DEGs related to melanogenesis, melanoma, cAMP, and Rap1 signaling pathways (Figure 4b,d). These results revealed that Butyroside D have a depigmentation effect by inhibiting pathways involved in melanogenesis stimulation.

### 2.6. Butyroside D Upregulated Oxidative Phosphorylation and Fatty Acid Elongation Associated GO and KEGG Pathways

Further analysis of the upregulated DEGs with Butyroside D was assessed. The GO analysis for both treatments was mostly focused on apoptotic process, oxidation reduction process, ATP, and the regulation of TOR signaling (Figure 4a,b). Studies have demonstrated the involvement of mTOR signaling in inducing hypopigmentation effects [40]. Consequently, this compound stimulated ATP content and, therefore, cell proliferation and melanogenesis inhibiting signaling.

The detailed upregulated genes enriched KEGG pathways in both conditions are related to fatty acid elongation, oxidative phosphorylation, and metabolic pathways (Figure 4b,d). Putting this all together, Butyroside D may present a mitochondrial biogenesis stimulating effect while exhibiting an anti-melanogenesis activity.

### 2.7. Classification of DEGs Regulated by Butyroside D

**Figure 5:**
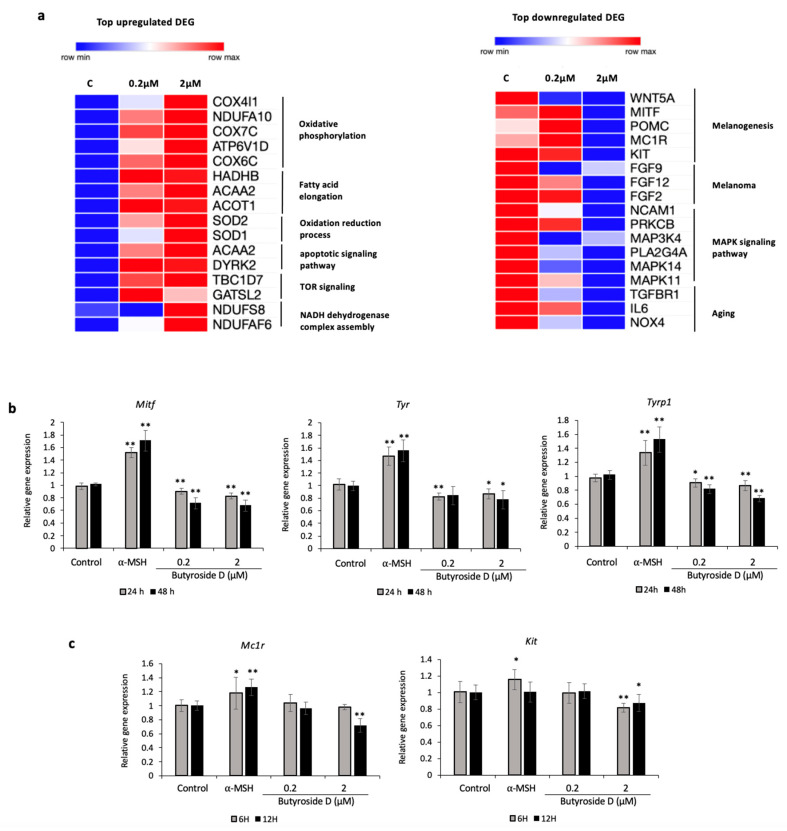
Butryoside D decreased the gene expression levels of melanogenesis-related genes in B16F10 cells. (**a**) Heatmaps displaying the relative expression levels of the top up- and downregulated genes by 0.2 and 2 μM Butryoside D. The related biological functions and pathways were also displayed. These heatmaps are generated by the online tool Morpheus Broad Institute. (**b**) Gene expression levels of Mitf, Tyr, Tyrp1, (24 h, 48 h) (**c**) Mc1r, and Kit (6 h, 12 h) were assessed using quantitative RT-PCR analysis. Results are presented as mean ± SD (*n* = 3). Statistical analysis was performed using one-way ANOVA where asterisks denote significant differences between the control and test groups being * *p* < 0.05 and ** *p* < 0.01.

In this section, we describe the top genes regulated by Butyroside D and the related pathways and functions involved. Two groups were formed for analysis, top upregulated DEGs, and top downregulated DEGs. Morpheus software version available online (https://software.broadinstitute.org/morpheus/) was used for the schematic representation and heat map generation (Figure 5a). The top upregulated genes formed 6 subgroups, the genes were related to oxidative phosphorylation (Cox4i1, Ndufa10, Cox7c, Atp6v1d and Cox6c), fatty acid elongation (Hadhb, Acaa2, Acot1), oxidation reduction process (Sod2, Sod1), apoptotic signaling pathway (Acaa2, Dyrk2), TOR signaling (Tbc1d7, Gatsl2), and NADH dehydrogenase complex assembly (Ndufs8, Ndufaf6). On the other hand, the top downregulated genes formed 4 subgroups and were related to melanogenesis (Wnt5a, Mitf, Pomc, Mc1r, Kit), melanoma (Fgf9, Fgf12, Fgf2), MAPK signaling pathway (Ncam1, Prkcb, Map3k4, Pla2g4a, Mapk14, Mapk11), and aging (Tgfbr1, Il6, Nox4). The analysis shows a clear distinction between the lowest concentration treatment of 0.2 μM Butyroside D and further differentiation of gene expression when increasing the treatment concentration to 2 μM using the same pure compound. Collectively, Butyroside D decreased melanogenesis-related genes and pathways, melanoma effectors, and aging mediators. On the other hand, it can be involved in stimulating ATP content and mitochondrial biogenesis.

### 2.8. Validation Assay of the Effect of Butyroside D on Melanogenesis Downregulation in B16F10 Cells

In order to confirm and validate the causal effect of Butyroside D on melanogenesis downregulation, we quantified the gene expression of pigmentation related enzymes Tyr and Tyrp1 [41], and the related transcription factor Mitf [42] upon 24 h or 48 h treatment exposure using RT-PCR. Figure 5b shows a significant decrease in mRNA levels of Mitf and Tyrp1 in a time dependent manner when B16F10 cells are exposed to either 0.2 μM or 2 μM Butyroside D compared to the control. The same reducing effect was observed in the case of Tyr gene expression; however, no significant difference between the two used concentrations was perceived (Figure 5b). Additionally, 2 μM Butyroside D was capable of the reduction of mRNA expression levels to significance of the two transcription factors Mc1r and Kit, highly involved in melanogenesis and melanocytes regulation [14], when compared to the control group after 6 h and 12 h of treatment (Figure 5c). This is consistent with microarray data shown in the Section 2.7 where the higher concentration of treatment exhibited a stronger activity on reducing melanogenesis related genes expression.

### 2.9. Effect of Butyroside D on Melanogenesis Downregulation in α-MSH Induced HEM Cells

**Figure 6:**
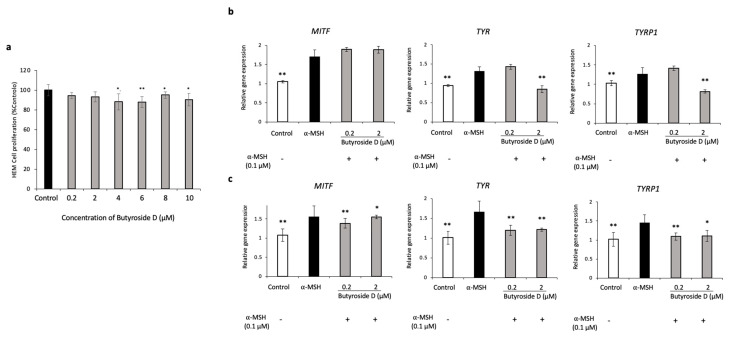
Validation of the effect of Butryoside D on the gene expression levels of melanogenesis-related genes using HEM cells. (**a**) MTT assay was performed to assess the cell proliferation of Butryoside D-treated HEM cells (0.2, 2, 4, 6, 8, and 10 μM) and cells were used to validate the gene expression levels of MITF, TYR, TYRP1, after (**b**) 24 h and (**c**) 48 h by quantitative RT-PCR, where melanin production was initially induced by α-MSH. Results are presented as mean ± SD (*n* = 3). Statistical analysis was performed using one-way ANOVA where asterisks denote significant differences between α-MSH group and control or test groups being * *p* < 0.05 and ** *p* < 0.01.

First, various concentrations of Butyroside D were applied to HEM cells for 48 h to check for cytotoxicity, if any. Data showed that the concentrations applied did not exhibit a toxic effect; however, the viability slightly decreased starting at 4 µM, thus for the following experiment, 0.2 and 2 µM Butyroside D were used (Figure 6a). It is worth mentioning that melanogenesis is initiated by the binding of α-MSH to MC1R in melanocytes [43]; thus, to investigate depigmentation-related effect, the cells should be pigmented in advance. For this purpose, we extended our study to determine the effect of Butyroside D application in HEM cells treated with α-MSH. Figure 6 shows a significant decrease in mRNA expression of MITF, TYR, and TYRP1 after 48 h treatment compared to α-MSH-treated group. Additionally, higher concentration of 2 µM Butyroside D significantly reduced the expression of TYR and TYRP1 compared to the α-MSH treated group after 24 h, whereas no significant effect was noted when using low concentration of 0.2 μM Butyroside D. MITF gene expression was not reduced after 24 h treatment with both studied treatment concentrations. Overall, Butyroside D showed a similar tendency on HEM cells in reducing the expression of the melanogenic enzymes, and their transcription factor, strengthening our previous finding on B16F10 cells.

## 3. Discussion

In the skin, melanocytes are found on the basal layer forming the epidermal melanin unit. The melanin is produced within the melanosomes of the melanocytes involving a series of signaling pathways [44]. In general, a mixed type of pheomelanin and eumelanin polymer is synthesized, leading to the variation in skin and hair color. The alteration of melanin production may cause considerable problems of esthetic nature, and it can vary from hyperpigmentation to depigmenting conditions; therefore, it may have a high impact on patients’ quality of life [45]. Hyperpigmentation is a skin condition where patches of skin become darker in color due to excess melanin production affecting all skin types [46]. Thus, it is important to explore treatment possibilities based on the prognosis to reduce the significant psychological impact and improve population healthy life, yet current therapies are still unfulfilled for skin pigmentation conditions [47]. Recently, there is a great demand for the development of safe and effective skin whitening agents [48], and several natural extracts have been proposed and identified to detain an inhibitory effect to melanin production [16,17,18]. In this current study, the effect of Butyroside D isolated from Argan press cake extract, previously reported to have a depigmentation activity, was extensively investigated in B16F10 cells and in human melanocytes (HEM) [29].

In melanogenesis studies, B16F10 cultured cells are widely used as a model for screening the anti-melanogenic effect of novel compounds [49]. After the confirmation of Butyroside D melanin reduction activity without affecting the cell viability on B16F10 cells, we proceeded with analyzing the impact of the treatment at low and high concentration (i.e., 0.2 μM and 2 μM) using DNA microarray technology. The biosynthesis of melanin is a result of a series of transformations and reactions engaging L-tyrosine mediated by the melanogenic enzymes under the influence of the transcription factor MITF, which is regulated by several signaling systems and transcription factors [50,51]. Thus, we investigated the effect of our sample on the up-stream related pathways controlling MITF. Our data showed a downregulation in the DEGs related to melanogenesis, cAMP, Wnt, and MAPK signaling pathways upon treatment with 2 μM Butyroside D. Furthermore, the pro-opiomelanocortin (Pomc) expression level was downregulated with the studied treatment of Butyrosiode D. The hormonal regulation of pigmentation is initiated with POMC proteolysis leading to melanotropins synthesis, namely α-, β-, and γ-MSH and ACTH. These melanotropins regulate pigmentation of many vertebrate species. The POMC level in skin is variable and is associated with physiological changes, UVR exposure, immune cytokine releases, and other cutaneous pathologies [52,53]. The role of cAMP pathway in melanin production and melanocyte survival is well-documented. Briefly, when α-MSH binds to the melanocytes master regulator MC1R, it induces cAMP activation, which in turn activates PKA to phosphorylate cAMP response element-binding protein (CREB) [54,55]. Consequently, CREB activates MITF, thus leading to the transcription of the melanogenesis-related enzymes TYR, TYRP1, and DCT [56]. Mitogen-activated protein kinase (MAPK) family proteins, such as p38, are well known for their important role in melanogenesis regulation; the phosphorylation of p38-MAPK increases the expression of MITF and TYR, resulting in the stimulation of pigmentation [57,58,59]. Another critical pathway regulating the melanogenesis and was negatively affected by Butyroside D treatment is the Wnt/β-catenin pathway. In fact, this pathway enhances MITF expression at the transcriptional level and controls the melanocytes from embryonic to maturation stages throughout their development, survival, and differentiation [59]. Finally, Butyroside D also upregulated pathways involved in stimulating ATP content and mitochondrial biogenesis, in particular oxidative phosphorylation involved in cell metabolism and energy yielding [60,61].

Accumulating evidence showed the strong interaction between canonical Wnt pathway and KIT signaling in regulating pigmentation [62]. KIT is pivotal in the signaling pathway controlling the stimulation of pigmentation, and mutations or deletion may cause melanin distribution in mammals skin and hair [63]. In addition, KIT plays an important role in the melanogenesis pathway. It was reported that this factor acts on melanocytes stem cells regulation, proliferation, and apoptosis [64]. Therefore, targeting these mentioned pathways can present a useful tool for depigmentation studies, which was the case upon Butyroside D applications. Even though microarray analysis results present interesting findings related to the overall Butyroside D anti-melanogenesis effect, the B16F10 model presents some limitations, bearing in mind the nature of murine melanoma cells that presents abnormal functions.

To further validate our microarray results, we assessed gene expression levels of two membrane receptors MC1R and c-Kit. Our results showed a significant downregulation in their expression when applying 2 μM Butyroside D after 6 h and 12 h treatment. Accordingly, we observed a decrease in the expression of Mitf and, therefore, the melanogenesis enzymes Tyr and Tyrp1 upon our treatment. We mentioned earlier in this paper, the importance of MC1R in triggering melanogenesis-related pathways; for this reason, this receptor is often established as one of the main dictators and a positive regulator of skin pigmentation [65,66]. The Butyroside D mechanism of action can be explained by inhibiting the binding between the receptor and the ligand leading to the repression of melanin biosynthesis-related pathway and, therefore, the depigmentation effect. However, at the skin level, differences between humans and other mammals do not reside on the chemical pathways of melanin production itself but rather the melanin transfer from the skin to the hair in case of mammals, which gives little color to the skin, in contrast to humans, where the melanin is transferred to keratinocytes and stays in the skin [66]. Therefore, human melanocytes make a unique model for further investigation.

Thus, to confirm whether Butyroside D acts specifically on the receptors, we co-treated human melanocytes with our sample and α-MSH, as the interaction between α-MSH-MC1R triggers the activation of the cAMP and, therefore, MITF and melanin production. We have found that the co-treatment significantly decreased the expression of MITF, TYR, and TYRP1 compared with only α-MSH. This finding can be supported by several studies where whitening compounds act on reducing α-MSH and MC1R binding and activation, which will eventually affect the transcription of MITF and TYR [67,68,69]. According to the current findings, it could be assumed that Butyroside D exerts an anti-melanogenesis effect on both pigment cells, B16F10 murine cells and human melanocytes. This compound specifically targets the inhibition of pigmentation-related pathways and the upstream effectors. Furthermore, the effect of Butyroside D may support the advancement and the development of an alternative class of therapeutics derived from a natural compound and its application in skin related research. Still, future research and molecular docking studies should be investigated to further explore the mechanism of action by which Butyroside D acts on melanogenesis. Ex vivo or co-culture systems, including melanocytes and keratinocytes, could be used to validate the Butyroside D effect in an environment that mimics the human skin. Finally, MC1R-targeted inhibition could substantially confirm the molecular interaction of this novel compound with melanocyte membrane receptor.

## 4. Materials and Methods

### 4.1. Plant Material

An 80% EtOH extract of Argan press cake (APC80-001, LOT NO. 20191105) was kindly provided by ADEKA CORPORATION, Tokyo, Japan.

### 4.2. General Experimental Procedures

The nuclear magnetic resonance (NMR) spectra were obtained with a JNM-ECS400 spectrometer (JEOL Ltd., Tokyo, Japan). The HRESI-MS data was recorded with a quadrupole time-of flight mass spectrometer (Agilent QTOF-LC-MS, Agilent Technologies, Santa Clara, CA, USA). Organic solvents were purchased from Wako Pure Chemical Industries (Osaka, Japan). Silica gel (75–120 mesh) and RP-C18 silica (38–63 μm) were purchased from Wako Pure Chemical Industries (Osaka, Japan). Biotage selekt (Uppsala, Sweden) equipped with NP-silica gel or RP-C18 flash columns chromatography were used for further fractionation. Purification of the isolated compounds was achieved by MPLC Pure C-850 Flash prep^®^ (Buchi, Flawil, Switzerland) with UV and ELSD detection and recycling preparative HPLC using a LaboAce LC-5060 system (Japan Analytical Industry, Osaka, Japan) equipped with Inertsil ODS-3, 5 μm, 20 × 250 mm, obtained from GL Sciences Inc., Tokyo, Japan. The analytical TLC was performed on precoated silica gel 60 GF254 (20 × 20 cm × 0.2 mm thick) or precoated RP-C18 F254 plates (5 × 7.5 cm × 0.2 mm thick) on aluminum sheets, both purchased from Merck Co., Darmstadt, Germany. The plates were developed using the appropriate solvent systems, and the developed chromatograms were examined under UV light 254 nm and 366 nm. The spots were made visible by spraying with vanillin/H_2_SO_4_ reagent before warming in an oven preheated to 110 °C for 5 min.

### 4.3. Butyroside D Extraction and Isolation Procedures

Ten grams of the 80% EtOH extract of Argan press cake was chromatographed on Biotage Selekt (Biotage, Uppsala, Sweden) (MPLC using DCM: MeOH (100:0→0:100) on a prepacked normal phase flash silica gel column Biotage^®^ Sfär HC Duo (Biotage, Uppsala, Sweden) 100 g with a flow rate of 120 mL/min to give 13 fractions (Fr. 1~13). Then, 800 mgs of Fr. 7 [eluted with DCM: MeOH (50:50)] was chromatographed on MPLC Pure C-850 Flash prep^®^ (Buchi, Flawil, Switzerland) using 0.1% FA/H_2_O: Acetonitrile (80:20→65:35) on a prepacked reversed phase flash C18 column (4 g) using ELSD and DAD (205, 210 nm) detectors to give 15 subfractions (Subfractions 1~15); 20 mgs of subfractions 6–8 [eluted with 0.1% FA/H_2_O: Acetonitrile (75:25)] was dissolved in 1 mL (0.1% FA/H_2_O: Acetonitrile (70:30) and applied to Inertsil ODS-3 column (5 μm, 20 × 250 mm, GL Sciences Inc., Japan) connected to recycling preparative LaboAce LC-5060 system (Japan Analytical Industry Co., Ltd., Japan) using a flow rate of 10 mL/min and RI and DAD detectors. The system was run with an isocratic elution of 0.1% FA/H_2_O: Acetonitrile (70:30). After one time recycling and manual collection of the resulting fractions (10 mL each), the target compound (7.8 mg) was isolated in a pure form. It has been identified as Butyroside D by comparing its physicochemical and spectral data with those reported in the literature [34].

### 4.4. Cell Culture

Murine B16F10 melanoma cell lines were obtained from Riken Cell Bank (Tsukuba, Japan) and maintained in Roswell Park Memorial Institute medium (RPMI 1640) (Gibco, MA, USA) supplemented with 10% foetal bovine serum (FBS) (Gibco, MA, USA) and 0.1% Penicillin/Streptomycin (Gibco, MA, USA). Human epidermal melanocytes (HEM) were obtained from Cell Applications, Inc. (San Diego, CA, USA) (Cat. No. 104-05n), maintained in melanocyte growth medium (Cell Applications, Inc., Tokyo, Japan). Both cell lines were maintained under sterile conditions and incubated at 37 ℃ in a 5% CO_2_ humidified incubator. The cell’s viability was determined using trypan blue exclusion method (Countess™, Thermo Fisher Scientific, Waltham, MA, USA).

### 4.5. Cell Proliferation Assay

The effect of Butryroside D on B16F10 or HEM cells proliferation was assessed with an MTT assay. Briefly, the cells were seeded into 96-well plates (5 × 10^4^ cells/well) and incubated for 24 h or 48 h, respectively, for B16F10 or HEM at 37 °C in a humidified atmosphere of 5% CO_2_. Growth mediums were then replaced with 90 μL/well of Butyroside D treatment diluted with RPMI 1640 medium or melanocyte growth medium with a concentration range of 0.2–10 μM kept for 48 h. Then, 10 μL of 3-(4,5-dimethylthiazolyl-2)-2,5-diphenyltetrazolium bromide (MTT) (Dojindo, Japan) (5 mg/mL) was added to each well and further incubated for 8 h. Lastly, 10% Sodium dodecyl sulfate (SDS) (Fujifilm Wako, Osaka, Japan), 100 μL/well, was added, and the plates were incubated overnight. The absorbance was measured at 570 nm using a microplate reader (Varioskan™ LUX, Thermo Fisher Scientific, Waltham, MA, USA).

### 4.6. Melanin Quantification

The release of melanin from the cells was determined as previously described with slight modifications [23]. Briefly, B16F10 cells were seeded and incubated overnight at 37 °C in a 5% CO_2_ atmosphere at a density of 5 × 10^4^ in 100 mm petri dish. The RPMI 1640 medium was then replaced with a fresh one in case of the control or Butyroside D (2 μM) diluted in the medium in case of target treatment or α-MSH (0.1 μM) for the positive control. After 48 h, the cells were harvested with trypsinization, and the viable cells were quantified using an automated cell counter. The cells were then solubilized using 0.1% Triton X-100 and precipitated using 10% trichloroacetate. Finally, the melanin was dissolved in 1 mL of 8 N NaOH and incubated for 2 h at 80 °C. The amount of melanin was determined after measuring the absorbance at 410 nm using Varioskan™ LUX. The melanin content was expressed as melanin content/cell (% of control).

### 4.7. RNA Extraction

The B16F10 cells or HEM cells were seeded at a density of 5 × 10^4^ or 1 × 10^5^, respectively, in a 6 well plate and incubated overnight. After the removal of the medium, the cells were treated with 0 µM, 0.2 μM or 2 μM of Butyroside D and α-MSH (0.1 µM) for 24 h and 48 h, in the case of B16F10 cells. However, HEM cells were treated with a combination of 0.1 µM α-MSH and 0.2 μM or 2 μM of Butyroside D to further confirm the effect of our target compound on melanin production in these cells. RNA extraction was performed using ISOGEN reagent (Nippon Gene, Tokyo, Japan) following the manufacturer’s instructions. RNA quantity and quality were determined using the NanoDrop 2000 spectrophotometer (ThermoScientific, Tokyo, Japan).

### 4.8. DNA Microarray

A microarray analysis was performed using Affymetrix’s GeneChip^®^ System (Affymetrix Inc., Santa Clara, CA, USA) using Mouse Clariom S Array following the manufacturer’s instructions. Briefly, the total RNA samples were prepared from the control group, 0.2 μM and 2 μM Butyroside D treated B16F10 cells (100 ng total RNA per sample) as described above. Next, complementary RNA (cRNA) was synthesized using IVT PLUS Reagent Kit (Affymetrix Inc., Santa Clara, CA, USA). The prepared samples were fragmented, labeled, and prepared for hybridization using a GeneChipTM Hybridization, Wash, and Strain Kit. Each specific sample was injected into the GeneChip TM cartridge of Clariom S Array and incubated with a rotation of 60 rpm for 16 h at 45 ℃ in the GeneChip^®^ hybridization oven 645. The arrays were washed and stained with GeneChip^®^ Fluidic station 450 and scanned with GeneChip^®^ Scanner 3000. After the normalization of raw image data using Expression Console Software (Affymetrix, Tokyo, Japan, URL: http://www.affymetrix.com), further gene expression analysis was performed using Transcriptome Analysis Console (TAC) software (Version 4, Thermofisher Inc., Tokyo, Japan). Differentially expressed genes (DEGs) were extracted based on the Fold change (FC) and *p*-values (FC > 1.5 and *p*-values < 0.05). All data supporting the findings of this study are included; we have deposited the microarray data into the Gene Expression Omnibus (GEO).

### 4.9. Real-Time Reverse Transcription Polymerase Chain Reaction (RT-PCR) Analysis

A SuperScript^TM^ IV VILO kit (Thermo Fisher Scientific, USA) was applied on the extracted RNA for first-strand cDNA synthesis according to the manufacturer’s instructions. Next, TaqMan primers and TaqMan Gene Expression Master Mix (Applied Biosystems, Foster City, CA, USA) were used for real-time quantitative PCR of the target gene on a 7500 Fast Real-Time PCR System (Applied Biosystems, Foster City, CA, USA). Microphthalmia-associated transcription factor (*Mitf*) (Mm00434954_m1), tyrosinase (*Tyr*) (Mm00495817_m1,), tyrosinase-related protein 1 (*Trp1*) (Mm00453201_m1), melanocortin 1 receptor (*Mc1r*) (Mm00434851_s1), kit oncogene (*Kit*) (Mm00445212_m1), and glyceraldehyde-3-phosphate dehydrogenase (Gapdh) (Mm99999915_g1) were purchased from Applied Biosystems. For the human primers, TYR (Hs00165976_m1), TYRP1 (Hs00167051_m1), MITF (Hs01117294_m1), and GAPDH (Hs02786624_g1) (Applied Biosystems, CA, United States) were used. The mRNA levels were quantified using Gapdh or GAPDH as a housekeeping endogenous control, and the expression levels were determined using the 2^−ΔΔCt^ method.

### 4.10. Statistical Analysis

All experiments were conducted in triplicate. The mean and standard deviation are reported in the current study. A one-way ANOVA was performed when the two groups were compared. A *p*-value of ≤ 0.05 was considered significant.

## Data Availability

The Supporting Data of this article can be found within the paper. The microarray data have been deposited in the NCBI GEO database. Expression data from 0.2 μM Butyroside D-treated B16F10. Available online: https://www.ncbi.nlm.nih.gov/geo/query/acc.cgi?acc=GSE213491 (accessed on 17 September 2022). Expression data from 2μM Butyroside D-treated B16F10. Available online: https://www.ncbi.nlm.nih.gov/geo/query/acc.cgi?acc=GSE213492 (accessed on 17 September 2022).

## Supplementary Materials

The following supporting information can be downloaded at: https://www.mdpi.com/article/10.3390/ijms232416021/s1.
