# Supplementary material for: New Butyroside D from Argan Press Cake Possess Anti-Melanogenesis Effect via MITF Downregulation in B16F10 and HEM Cells
Source: Int J Mol Sci. 2022 Dec 16;23(24):16021. doi: 10.3390/ijms232416021 (PMC9785346; doi:10.3390/ijms232416021)
Supplement: Supplementary file 1 [file ijms-23-16021-s001.zip › ijms-2063890-supplementary.pdf]

Butyroside D

White amorphous powder, $[\alpha]_{D}^{25}$ - 22.4 (*c* 0.86, EtOH).

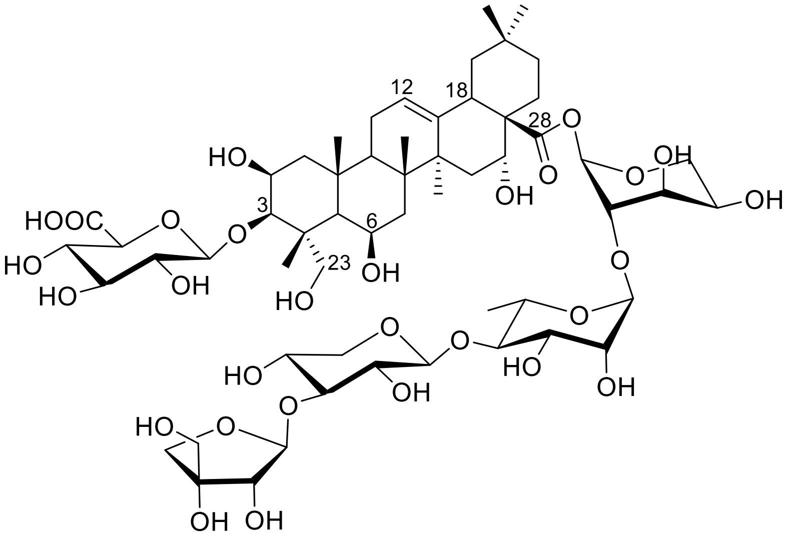

Chemical Formula: $C_{57}H_{90}O_{29}$
Exact Mass: 1238.5568

Structure of Butyroside D

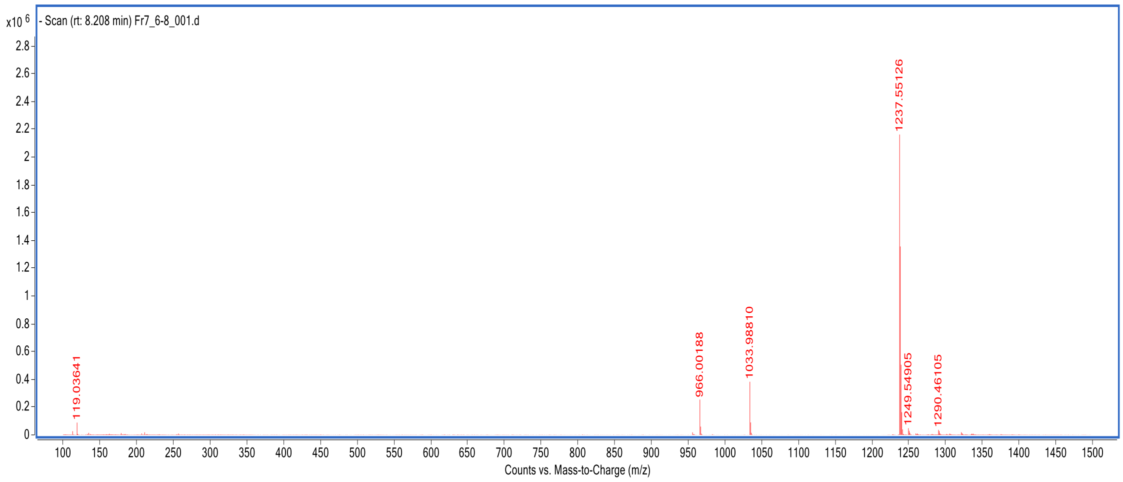

HR-ESI-MS of Butyroside D

- Measured [M-H]$^-$ 1237.5513
- Calculated [M-H]$^-$ 1237.5490

Δ = 0.0023

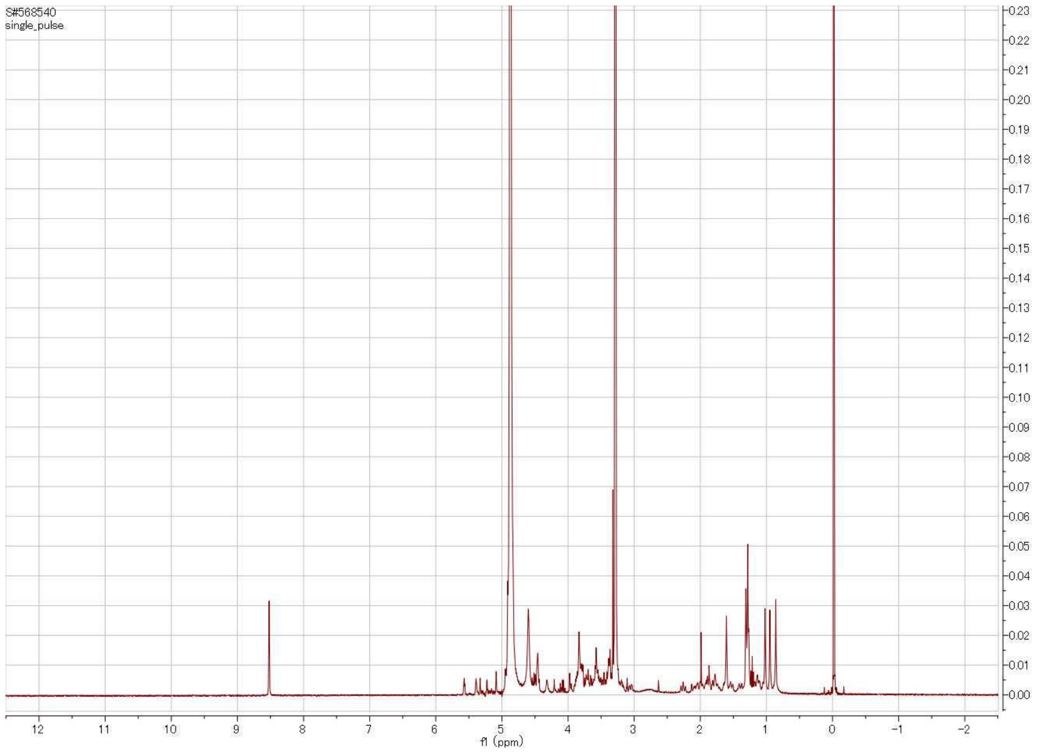

^{1}H-NMR analysis of Butyroside D (CD_3OD, 400 MHz)

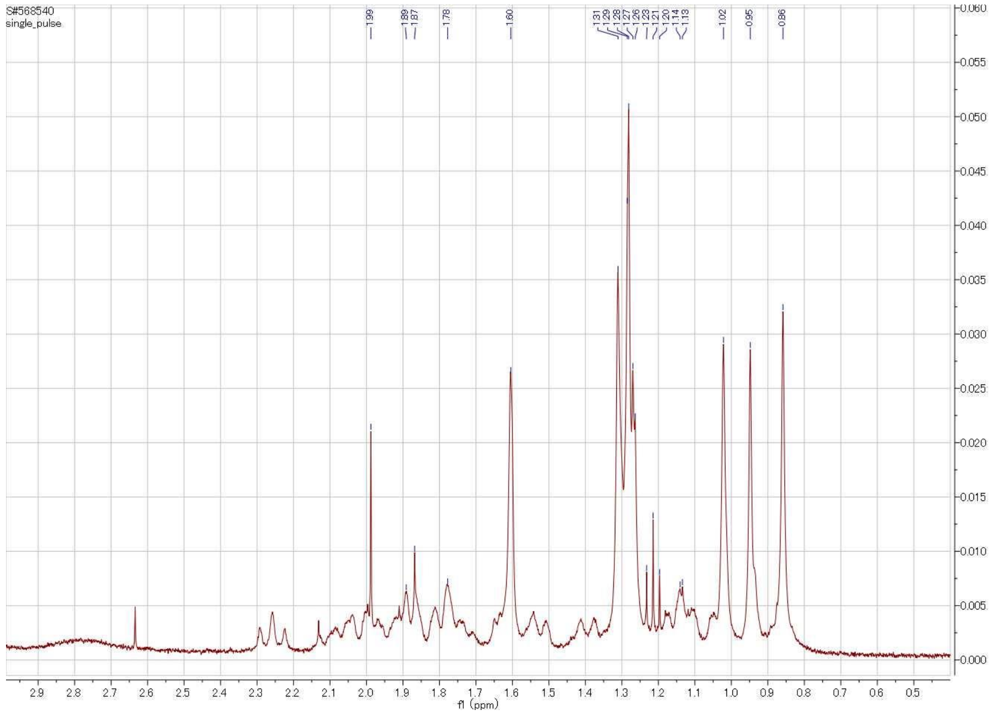

^{1}H-NMR (Expansion) analysis of Butyroside D (CD_3OD, 400 MHz)

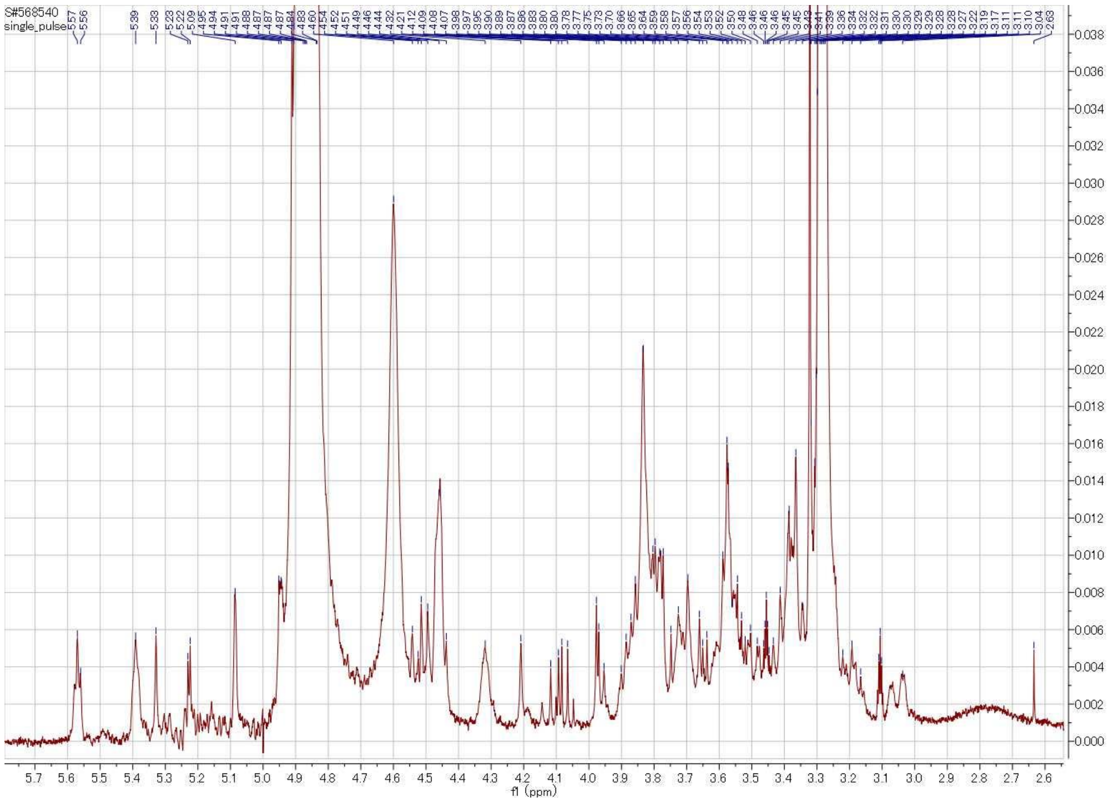

^{1}H-NMR (Expansion) analysis of Butyroside D (CD_3OD, 400 MHz)
